# Skewed Epigenetics: An Alternative Therapeutic Option for Diabetes Complications

**DOI:** 10.1155/2015/373708

**Published:** 2015-04-30

**Authors:** Gabriele Togliatto, Patrizia Dentelli, Maria Felice Brizzi

**Affiliations:** Department of Medical Sciences, University of Turin, Corso Dogliotti 14, 10126 Turin, Italy

## Abstract

Vascular complications are major causes of morbidity and mortality in type 2 diabetes patients. Mitochondrial reactive oxygen species (ROS) generation and a lack of efficient antioxidant machinery, a result of hyperglycaemia, mainly contribute to this problem. Although advances in therapy have significantly reduced both morbidity and mortality in diabetic individuals, diabetes-associated vascular complications are still one of the most challenging health problems worldwide. New healing options are urgently needed as current therapeutics are failing to improve long-term outcomes. Particular effort has recently been devoted to understanding the functional relationship between chromatin structure regulation and the persistent change in gene expression which is driven by hyperglycaemia and which accounts for long-lasting diabetic complications. A detailed investigation into epigenetic chromatin modifications in type 2 diabetes is underway. This will be particularly useful in the design of mechanism-based therapeutics which interfere with long-lasting activating epigenetics and improve patient outcomes. We herein provide an overview of the most relevant mechanisms that account for hyperglycaemia-induced changes in chromatin structure; the most relevant mechanism is called “metabolic memory.”

## 1. Introduction

Diabetes is a leading cause of morbidity and mortality across the world [1, 2]. Population age, obesity, and modern sedentary lifestyles mean that the incidence of type 2 diabetes (T2D) has significantly increased worldwide in recent years [2–4]. Type 2 diabetes is associated with long-term vascular complications, including endothelial dysfunction, atherosclerosis, nephropathy, retinopathy, and peripheral arterial disease (PAD) [5–8]. Although advances in therapy have led to a significant reduction in both micro- and macrovascular complications, overall cardiovascular risk is still a clinical problem [9–12]. The Diabetes Control and Complications Trial (DCCT/EDIC) [9, 10] and the UK Prospective Diabetes Study (UKPDS) [11, 12] have shown that a coupling of intensive glycaemic control and the close monitoring of both blood pressure and cholesterol may not be effective in protecting patients from vascular complications, thus suggesting that diabetes-associated cardiovascular risk factors may rely on the so-called “metabolic memory.” “Metabolic memory” mainly reflects epigenetic changes driven by hyperglycaemia-induced oxidative stress [13–16]. Attention has so far been paid to dissecting these epigenetic mechanisms and defining a diabetes epigenetic signature which would allow new therapeutic approaches, which could “erase” epigenetics and improve patient outcomes, to be developed. This concise review provides a synopsis of how epigenetic mechanisms impact long-term diabetes-associated complications.

## 2. Epigenetic Changes: ROS-Mediated Effects in Diabetes

Free radical accumulation in the vasculature of diabetic patients is considered to be the most significant mechanism in long-lasting vascular complications [17, 18]. The key role played by mitochondrial ROS generation is a key pathway in the natural history of diabetes-associated vascular disease and has been documented by the seminal studies of Brownlee and colleagues [18–21]. Similarly, data provided by El-Osta and colleagues have contributed to current knowledge that links ROS of mitochondrial origin to endothelial dysfunction [22–24]. More recently, the intimate association between increased oxidative stress of mitochondrial origin and defective antioxidant machinery has been used to explain pathophysiological pathways associated with a prolonged response even to transient hyperglycaemia exposure [16, 25]. The fact that this hyperglycaemic environment can be remembered by the vasculature, first suggested by Roy and colleagues in 1990 [26], has recently furnished the mechanistic elucidation of what is called “metabolic memory.” As a matter of fact “metabolic memory” refers to epigenetic changes driven by hyperglycaemia-mediated mitochondrial ROS production. The term “epigenetic” was first coined by Waddington in 1942 to explain how environmental factors can affect the phenotype without altering the genotype. Although its conceptual origins date back to Aristotle (384–322 BC), who believed in “epigenesis” in the development of an individual organic form from the unformed, traces of epigenetics were seen in the literature by the mid-nineteenth century. It currently refers to heritable traits that do not match changes in the DNA sequence. This concept has recently been cited as a means to explain how the complex interaction between genotype and environmental cues (hyperglycaemia in diabetes) brings about “metabolic memory” [13–16, 22–25]. Long- (heritable) or short-acting (nonheritable) environmental cues can lead to long- or short-term epigenetic effects, respectively. In diabetes, both mechanisms can take part in reprogramming the epigenome and eventually give details about the awful “metabolic memory” of postprandial hyperglycaemia episodes [14, 15, 27]. Over the last few years, a number of studies have shown that chromatin modification, in the nucleus, and microRNA (miR) deregulation, in the cytoplasm, are hallmarks of the long-lasting detrimental effects of hyperglycaemia which may translate into the high residual cardiovascular risk that diabetic patients face despite multifactor intervention [28].

## 3. Epigenetics in the Nucleus

Chromatin is the form in which the nucleic acids are found in the cell. Chromatin is, in fact, a macromolecular complex formed of DNA, proteins (histones), and RNA. Gene transcription is strictly controlled by the dynamic arrangement of chromatin: transcriptionally active (euchromatin) and transcriptionally inactive (heterochromatin). Histone posttranslational modifications (PTHMs) and DNA methylation both control gene expression. Changes in chromatin structure as a result of an imbalance in ROS production is the most relevant nuclear epigenetic mechanism and accounts for the long-lasting effects of hyperglycaemia [29–31]. Histones and DNA are considered to be the foremost targets of epigenetic factors in this setting [29–31]. PTHM modification alters chromatin packing and results in different DNA arrangements and readouts. The most relevant PTHM changes in diabetes are lysine acetylation, by histone acetyltransferases (HATs), and lysine and arginine methylation, by histone methyltransferases (HMTs) [32]. The acetylation of H3K and H4K terminal tails generally erases any interaction with DNA, allowing euchromatin to form and gene transcription to occur. Conversely, histone methylation is associated with a more complex and “capricious” gene transcriptional status [13–16]. Indeed, the mono-, di-, or trimethylation of various lysine residues in the H3 tail can cause either transcriptional activation or repression. Of the enzymes that regulate this process, histone methyltransferases Set7 and Set9 are the most relevant, are activated in the hyperglycaemic settings, and mainly control genes involved in the inflammatory response [33]. Nevertheless, as a result of histone acetylation/methylation, a number of inflammatory genes undergo transcription in response to hyperglycaemia over time [14, 16, 28]. This sustains the relevance of such a mechanism in perpetuating tissue damaging signals in diabetes. Even transient hyperglycaemia can lead to long-lasting epigenetic changes in the promoter of the NF-*κ*B-p65 gene, resulting in the increased expression of its product in the diabetic setting [22, 34]. More recently, it has been reported that the mitochondrial adaptor protein p66^shc^ may be transcriptionally regulated by H3 acetylation [25]. p66^shc^ overexpression and mitochondrial translocation have been linked to increased mitochondrial ROS generation, reduced nitric oxide bioavailability, and vascular cell apoptosis [25]. The biological relevance of H3 acetylation in p66^shc^ expression was further supported by the observation that the overexpression of one member of class III histone deacyltransferase (HDAC) SIRT1 inhibits hyperglycaemia-induced p66^shc^ expression, improves endothelial function, and reduces oxidative stress markers [35] A close connection between p66^shc^, SIRT1, and tumour transcription factor p53 [25, 36, 37] has been also reported, which supports the crucial role that the epigenetic mechanism plays in dictating cell fate, predominantly in the vascular system. p66^shc^ is a good example of a nuclear epigenetic target as its expression can be regulated by DNA methylation as well [25]. DNA methylation depends on three different DNA methyltransferases (DMT1, DM3A, and DM3B) in mammalian cells [38]. In these cells, DMT3A and DMT3B provide* de novo* methylation of the CpG dinucleotides on cytosine [39]. Therefore, DNA regions with increased CpG clusters, also called “CpG islands,” dictate gene transcription status [40, 41]. The hypermethylation of CpG islands in the promoter region commonly translates into transcription repression, while low methylation has the opposite effect [42]. The fact that DNA methylation can influence the expression of genes involved in diabetes-associated complications has been documented in preclinical models of nephropathy and retinopathy [43, 44]. In addition, genome-wide methylation studies indicate that 19 prospective CpG regions are associated with an increased risk of diabetic nephropathy in type 1 diabetic patients [45]. Finally, studies performed on peripheral blood cells recovered from patients participating in the DCCT/EDIC study have demonstrated a correlation between chronic complications and H3K9 acetylation [46].

## 4. Epigenetics Regulated by Cytoplasmic Factors

In addition to DNA methylation and histone modifications, microRNAs (miRs) have emerged as relevant epigenetic regulators. miRs are a class of noncoding sequences which regulate gene expression at the posttranscriptional level via the inhibition of ribosome function, resulting in the translational repression or degradation of the target mRNA [47]. Although miRs most commonly lead to decreased gene expression, they can also induce gene upregulation by negatively modulating the expression of inhibitory genes. However, miR downregulation may also upregulate previously suppressed genes [47–49]. More importantly, single miR targets multiple genes while single genes can be regulated by several miRs [47–49]. A number of studies demonstrating that miR expression is tissue specific have suggested that miRs may be important to establish and maintain cell type and tissue identity [49–53]. However, in recent years their role in mediating chronic diseases has been deeply investigated. As a matter of fact, abnormal miR levels have been described in clinical and preclinical models of diabetes-associated complications [54, 55]. The role of miRs in diabetic nephropathy (DN) has been extensively studied. Altered levels of miR25- and miR21-driven posttranscriptional regulation of the NADPH-oxidase subunits NOX4 and PTEN, respectively, have been linked to diabetic nephropathy in rat and mouse models [56, 57]. Moreover, Wang and colleagues [58] demonstrated that the treatment of proximal-tubular epithelial cells with TGF-*β*1 and TGF-*β*2 led to miR-141 and miR-200a downregulation. The overexpression of miR-200a was found to be associated with the posttranscriptional regulation of TGF-*β*2 which is consistent with miR-200a's role in the development and progression of TGF-*β*-dependent kidney fibrosis [58]. Similarly, increased miR-377 levels contributed to fibronectin production and kidney fibrosis in human and mouse mesangial cells that were exposed to TGF-*β* and hyperglycemia [59]. Furthermore, kidney specific miR-192 knockdown was found to provide protection against diabetic nephropathy-associated kidney fibrosis [60].

Several miRs have been associated with the early stages of diabetic retinopathy (DR) development as well as disease progression. An evaluation of retinal endothelial cells and diabetic retinas indicated that numerous different targets were involved in angiogenesis, inflammation, and oxidative stress. Feng et al. [61] demonstrated that both endothelial cells from large vessels and retinal microvessels that had been treated with high glucose content express high fibronectin (FN) levels by means of miR-146a downregulation. Moreover, they also show that miR-200b controls miR-146a and FN expression, via the histone acetylator p300, in hyperglycemic conditions not only in the retina but also in the heart and kidney.

Recent data indicate that the reality is more complicated than expected as other miRs are involved in HDAC regulation [62]. Indeed, it has been reported that miR-29 and miR206 overexpression leads to HDAC4 translational repression during myogenic differentiation [63]. Furthermore, Lin et al. [64] demonstrated that hyperglycemia contributes to nephrin acetylation and renal dysfunction by impairing miR-29a signaling which resulted in improved HDAC4 action. Various miRs, including miR1, miR206, miR133a, miR221/222, and miR126, have also been implicated in cardiovascular complications as they act on various targets in cardiomyocytes and vascular cells [17, 65–67], while cardiac microvascular endothelial cells were found to express high miR-320 levels in a preclinical model of type 2 diabetes [68].

Although not as intensely investigated, unbalanced ROS production was found to be involved in miR deregulation [69]. In this regard, we have recently reported that interfering with mechanisms involved in ROS production rescues miR126 expression and reverts the epigenetic pattern in endothelial cells [70]. Indeed, we were able to reprogram some epigenetic abnormalities, such as SIRT1 expression and p53 and H3K56 acetylation status, in ob/ob mice subjected to peripheral artery disease by administering the naturally occurring hormone, unacylated ghrelin (UnAG) [70]. As this effect translates into hind limb function improvement, the notion of epigenome reprogramming being able to offer clinical benefits in a diabetic setting is strengthened.

## 5. An Epigenomic Fingerprint for Identifying High-Risk Diabetic Patients

As stated above, the epigenome refers to DNA methylation, histone modification, and chromatin accessibility throughout the genome. Each cell type possesses a unique epigenome, which defines its regulatory program, and each individual displays unique epigenome modifications [71, 72]. It has been extensively demonstrated that exposure to environmental cues affects an individual via their own epigenome. It has therefore become clear that an understanding of how genetic, epigenetic, and environmental factors interact with each other to drive chronic disease development and progression is mandatory [14, 15, 27, 28, 73]. Advances in genome-wide technologies and bioinformatics have opened up new possibilities in recent years [74, 75]. The use of chip-sequencing to map several histone and DNA methylation marks has demonstrated that changes in DNA methylation patterns and histone modification are crucial for genetic information readout [76]. A genome-wide map of epigenetic modifications across several pathologic conditions, including diabetes, would therefore be particularly relevant in therapeutic target identification. The Human Epigenome Project, established in 1999, identifies and interprets genome-wide DNA methylation patterns in human genes. It is therefore widely expected that an exact and detailed map of epigenetic variations may allow scientists to better understand epigenetic biogenesis and function and further improve gene-based treatment as well as preventing diabetic complications. In this sense, epigenomic analysis in human pancreatic islets shows a strict association with gene expression and histone modification and is possibly relevant to diabetes [77]. More recently, data from Nilsson et al. [78] have provided support for the role played by DNA methylation in modulating the expression of genes associated with discrete pathways that are causally involved in T2D, thus strengthening the notion that genetics, epigenetics, and environment cues may also mutually contribute to type 2 diabetes susceptibility. A challenge for the future thus lies in using next-generation sequencing techniques, such as RNA-sequencing and whole-genome sequencing, to identify the gene-expression regulatory network associated with high-risk diabetic patients on the DNA level.

## 6. Pharmacological Reprogramming of the Epigenome: A Novel Therapeutic Option

A detailed characterization of deranged gene expression patterns in diabetes has pulled clinicians towards epigenetic reprogramming approaches. A number of preclinical studies have indicated that gene expression can be modulated by treatment which interferes with the acetylation and methylation of histone/DNA complexes [16, 28]. As a matter of fact, folate treatment can affect the expression of genes regulated by CpG methylation by reducing homocysteine [79]. p66^shc^ is one of the genes regulated by CpG methylation in response to homocysteine [80]. A complete exploration of the potential clinical applications of folate is therefore an interesting challenge for the future. Other therapeutic epigenome-based options have been proposed; however, potential clinical application is still being debated. A reason for this was the failure of resveratrol, which was originally described as a potent SIRT1 activator that was able to rescue NO bioavailability [81] and increase p66^shc^ promoter deacetylation [35], to provide beneficial effects when supplemented in obese patients [82]. A number of preclinical studies have also been performed on metformin, which is currently used to treat diabetic patients and which is known to increase SIRT1 expression and suppress NF-*κ*B activation [37]. Indeed, it has been shown that metformin is effective in suppressing the deleterious effects of hyperglycaemia in retinal capillary endothelial cells and in the retinas of diabetic animals by modulating the SIRT-1/LKB1/AMPK pathway [37]. However, the early enthusiasm for this drug that acts in the metabolism is in stark contrast with the controversial results obtained in UKPDS and Action to Control Cardiovascular Risk in Diabetes (ACCORD) trials, where metformin treatment led to reduced cardiovascular events in the first and an increased occurrence of these events in the second. Finally, the use of curcumin, known to exert beneficial effects by acting on the histone acetylator p300, has been proposed. However, even curcumin failed to be successful and the authors suggest that curcumin finds use as an adjuvant therapy in type 2 diabetic patients [83].

While the removal of epigenetic tags appears to be crucial in improving diabetes patient outcomes, clinical study results are far from convincing. It is, however, always worth remembering that the number of trials and the patients included in them is still a source of major bias when attempting to obtain persuasive results. This implies that effort should be made to assess the “true” clinical efficacy of the above compounds in randomized controlled trials. Moreover, genome-wide analysis of DNA methylation and histone modifications may well be the tool of the future when exploring new “personalized signature” based therapeutics.

## 7. Conclusions and Perspectives

**Figure 1:**
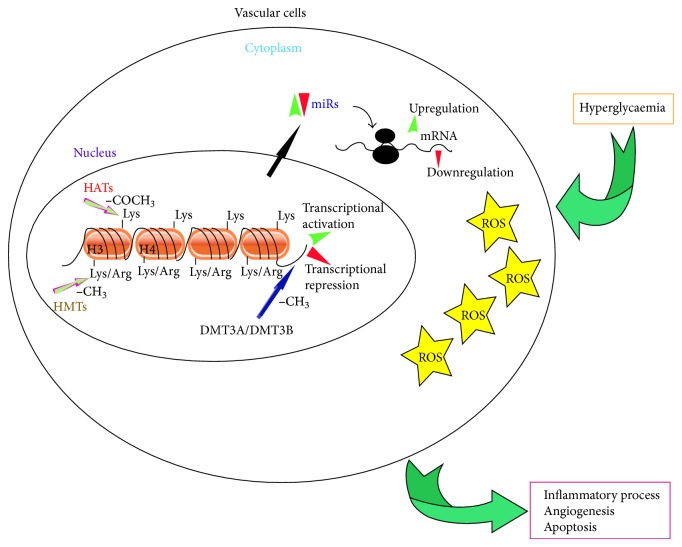
Schematic representation of epigenetic mechanisms in diabetic setting. In the nucleus ROS-mediated histone acetylation/methylation as well as DNA methylation accounts for gene transcriptional activation or repression. In the cytoplasm ROS-mediated changes in miR expression drive posttranscriptional regulation of target genes. Such epigenetic changes impact long-term diabetes-associated complications. HTAs: histone acetyltransferases; HMTs: histone methyltransferases; DMT: DNA methyltransferase; ROS: reactive oxygen species; miRs: microRNAs; Lys: lysine; Arg: arginine; –COCH_3_: acetyl radical; –CH_3_: methyl radical.

Increased ROS of mitochondrial origin and defective mitochondrial electron transfer chains appear to be the most relevant mechanisms in what is known as “metabolic memory.” “Metabolic memory,” which refers to the effects of hyperglycaemia on long-term vascular complications, mainly relies on changes in chromatin structures and miR expression (Figure 1) [28]. Over the last few years, more details on how hyperglycaemia drives changes in the epigenome have been provided and a putative epigenome signature has been proposed. Moreover, genome-wide methylation studies have indicated that epigenetic mechanisms can predict nephropathy in type 1 diabetic patients [45]. Thus, future efforts should be directed towards clinically exploiting the epigenome signature so as to identify patients with a high risk of complications and epigenome targeting approaches. Indeed, recent efforts have been directed towards developing agents which are able to reprogram the epigenome and hamper the vicious hyperglycaemia driven circle which leads to residual cardiovascular risk in diabetic patients [84]. However, it is always worth remembering that since environmental factors are the main triggers of epigenetic modifications, a suitable lifestyle should be recommended above all other factors.
